# Finding Optimal Thermo-Mechanical Processing for a TNTZ-O Beta-Titanium Alloy

**DOI:** 10.3390/ma18235281

**Published:** 2025-11-23

**Authors:** Vasile Danut Cojocaru, Anna Nocivin, Doina Raducanu, Claudia Ioana Cojocaru, Raluca Elena Irimescu, Mihai Bogdan Galbinasu

**Affiliations:** 1 Department of Metallic Materials Processing and Environmental Engineering, National University of Science and Technology POLITEHNICA of Bucharest, 060042 Bucharest, Romania; dan.cojocaru@upb.ro (V.D.C.); doina.raducanu@upb.ro (D.R.); claudia.cojocaru@stud.sim.upb.ro (C.I.C.); raluca.irimescu@stud.sim.upb.ro (R.E.I.); 2 Faculty of Mechanical, Industrial and Maritime Engineering, Ovidius University of Constanța, 900527 Constanța, Romania; 3 Dental Medicine Faculty, University of Medicine and Pharmacy “Carol Davila” Bucharest, 020021 Bucharest, Romania; bogdan.galbinasu@umfcd.ro

**Keywords:** β-titanium alloys, XRD, SEM, tensile tests, mechanical properties

## Abstract

A titanium-based alloy, with niobium, tantalum and zirconium as its main alloying elements, and a small amount of oxygen, was subjected to a thermo-mechanical processing program consisting of hot and cold rolling interspersed with different solution treatments applied above the β-transus temperature. The objective was to analyze the correlation between structure, processing methods and resulting mechanical properties in order to find an appropriate optimized processing path to obtain suitable mechanical characteristics for application in hard tissue implantology. By using microstructural analysis, such as X-ray diffraction and scanning electron microscopy, a series of microstructural features were analyzed. The enthalpy of mixing—ΔHmix—and the atom size difference parameter—δ—were determined, through which the β-solid solution stability was estimated. Tensile tests were performed, through which the main mechanical characteristics were determined: a yield strength (0.2%) and ultimate tensile strength of around 550 MPa and 650 MPa, respectively, with a low Young’s modulus of 56–58 GPa and an improved elongation to fracture value of 17–18%, associated with a good mechanical osteo-compatibility parameter.

## 1. Introduction

Titanium alloys are widely used in orthopedic applications [1,2,3], for which two aspects must be considered: the alloying elements used, which must be biocompatible with the human body [4,5], especially for long-term bone implants, and the methods of processing the alloy into finished shapes, so that the obtained mechanical properties can be correlated with the surrounding bone elements [6,7,8].

Regarding selecting the appropriate alloying elements, the Ti–Nb–Ta–Zr (TNTZ) combination presents the most appreciated characteristics for orthopedic applications [9,10,11,12], motivated by their high biocompatibility [13] coupled with high corrosion resistance in the human body environment [14,15].

The processing methods must involve easier and faster mechanical processing to obtain appropriate shapes with satisfactory mechanical strength characteristics [16,17]. In general, the TNTZ system has not been investigated very deeply in the literature in terms of correlations between processing, structure and mechanical properties—[4,5,6,13], but rather biocompatibility assessments of these alloys have been conducted. To date, the metastable β-type structures of titanium alloys have been proven to have the most accessible mechanical processability due to the β phase bcc structure [18,19]. As the main β-stabilizing elements [20], niobium and tantalum are also highly appreciated for their biocompatibility also [13]. Niobium, for example, acts as an effective β-stabilizing element, if added in a proportion of approximately 30–40% g. [21].

Zirconium, another appreciated biocompatible alloying element [13], is proven to have an inert, even negligible, effect on β phase and α phase stability as well [22,23,24]. However, when added to an alloy together with β-stabilizing elements such as tantalum and especially niobium, it can also contribute to stabilizing the β phase, concomitantly increasing toughness and reducing the Young’s modulus of Ti alloys [25]. Zirconium can also prevent the ω phase formation, which is known to seriously weaken the mechanical properties of titanium alloys [23,24,25].

For metastable β-titanium alloys, the most sensitive issue is that, although they have easy and fast mechanical processability due to β-phase bcc crystal structure ductility [4,14,19,26], their mechanical strengths do not reach the values required for bone implants [26,27]. For example, for single-phase β TNTZ alloys, the 0.2% yield strength (YS) reported to date is unfortunately only about 350–500 MPa [28,29,30,31], with half of the values corresponding to the Ti-6Al-4V alloy [29]. Therefore, there is a legitimate need to increase the yield strength for these β-type TNTZ alloys, while decreasing the Young’s modulus to values as close as possible to those of cortical bone (~30 GPa) [1,4,10].

To achieve this, the challenge is finding an appropriate balance between two apparently dichotomous actions: on the one hand, there is a series of plastic deformations, even severe plastic deformations, through which a significant reduction in grain size can be achieved, thus substantially increasing the yield strength; on the other hand, it is a question of inserting a series of solution treatments (STs) between the plastic deformations, which can ensure adequate ductility, while maintaining, as much is possible, the high strength level already obtained through plastic deformations.

Another possible option for increasing strength, recently studied and reported in various articles [32,33,34], concerns adding a very small amount of oxygen (generally less than 0.3 wt.%) to TNTZ alloys; as a strong interstitial strengthening element of the β phase [35,36,37], it can act as a β-stabilizer and even as an inhibitor stress-induced martensitic phase (α″) formation [38,39].

Although some results of both acting routes—thermo-mechanical treatments and oxygen addition—are recorded [21,36,37,40,41,42,43], there is no standard procedure that can be successfully applied to TNTZ alloys. All of these experiments involve a sensitive balance between ductility and strength, which depends significantly on both the alloy’s chemical composition and the chosen processing scheme. For each TNTZ alloy, many challenging experimental programs can still be carried out with optimized processing schemes that imply optimized experimental parameters.

Therefore, for the present research program, our aim was to find an appropriate thermo-mechanical processing route for an oxygen-doped Ti–Nb–Zr–Ta alloy to achieve a good balance between low Young’s modulus and high strength, thereby creating a suitable implant material.

## 2. Materials and Methods

### 2.1. Alloy Synthesis and Thermo-Mechanical Processing

The studied titanium alloy had the nominal composition Ti-36.5Nb-4.5Zr-3Ta-0.16O (wt.%). Niobium was included at a relatively high amount for β-phase stabilization, while Ta and Zr were added for biocompatibility. A small amount of oxygen (~0.16 wt.%) was used to improve strength.

The alloy was produced by levitation induction melting (FIVE CELES-MP25, Five’s Group, Lyon, France) under high vacuum (10^−4^–10^−5^ mbar) using high-purity elements (Ti ≥ 99.7%, Nb ≥ 99.9%, Zr ≥ 99.6%, Ta ≥ 99.9%). For the oxygen, a Ti–Nb pre-alloy with controlled oxygen content was used. The ingots were re-melted several times to ensure chemical homogeneity, with EDS used to confirm a composition close to the nominal one. Specimens (2 × 15 × 45 mm) were cut using a Metkon MICRACUT 200 machine (Metkon Instruments Inc., Gebze, Turkey).

**Figure 1:**
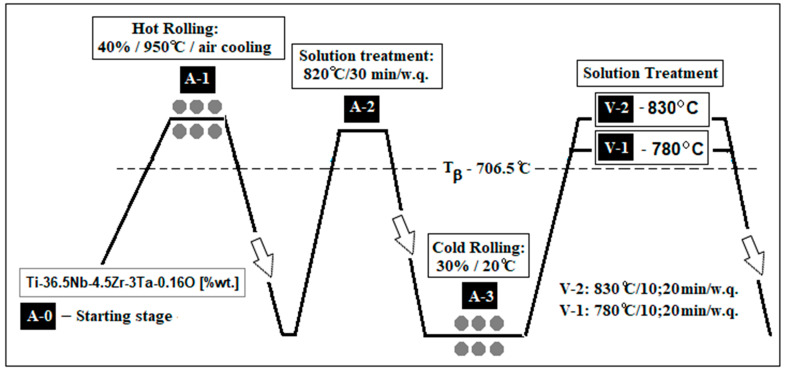
The thermo-mechanical processing route of the Ti-36.5Nb-4.5Zr-3Ta-0.16O (wt.%) alloy: A-0—the as-cast alloy; A-1—the hot rolled alloy (ε = 40% at 950 °C/air cooling); A-2—the solution treated alloy (820 °C/30 min/water quenching); A-3—the cold rolled alloy (ε = 30% at 20 °C); V-1—variant 1 of final solution treatment (780 °C/10 and 20 min/water quenching); V-2—variant 2 of final solution treatment (830 °C/10 and 20 min/water quenching).

The processing schedule (Figure 1) comprised preliminary stages (A) followed by two solution treatment variants (V1 and V2).

To find the β-transus temperature, the empirical calculation proposed by Sun et al. [44] was used, which is more comprehensive than the other proposed calculation [45] as it refers to 19 possible alloying elements, including those in the present case. Thus, the calculated β-transus temperature for the studied alloy results was 706.5 °C.

- Preliminary stages (A):
  - A-0: Starting Stage: as-cast sample;
  - A-1: Hot Rolling (40% reduction at 950 °C, air cooled-a.c.);
  - A-2: Solution Treatment (820 °C, 30 min, water quench-w.q.);
  - A-3: Cold Rolling (30% reduction at room temperature).

- Solution Treatments (V):
  - V-1: Near β-transus (780 °C); V-1-1 for 10 min.; V-1-2 for 20 min.
  - V-2: Above β-transus (830 °C); V-2-1 for 10 min.; V-2-2 for 20 min.
  - Holding times: 10 and 20 min, followed by water quenching.

Rolling was performed on a Mario di Maio LQR120AS mill (3 m/min), Mario di Maio Inc., Milan, Italy, while solution treatments were conducted in a high-vacuum GERO SR 100 × 500 furnace (Carbolite-Gero, Neuhausen, Germany). These processes aimed to optimize mechanical behavior for potential biomedical use.

### 2.2. Microstructural and Mechanical Characterization

Phase identification was performed using X-ray diffraction (Philips PW 3710, Cu k-α, 30°–90°, 2θ° range, Philips, Eindhoven, The Netherlands), while MAUD v2.33 and PeakFit v4.12 software were used for pattern simulation and peak analysis.

Microstructural observations were made using a TESCAN VEGA II-XMU SEM with a BRUKER eFlash 1000 EBSD detector (Tescan Orsay, Brno, Czech Republic). Samples were sectioned (RD-ND plane; RD—rolling direction; ND—normal direction), mounted in epoxy, ground and polished using diamond suspensions and colloidal silica.

Tensile tests were carried out on a Gatan Micro Test-2000N system (Gatan Inc., Pleasanton, CA, USA) at a strain rate of 1 × 10^−4^ s^−1^ using dog-bone samples (0.8 × 6 × 20 mm). The yield strength (0.2%), ultimate tensile strength (UTS), elongation to fracture (ε_f_) and elastic modulus (E) were determined. Mechanical osteo-compatibility was assessed as the ratio YS/E.

## 3. Results

### 3.1. Microstructural Characterization of the Studied Alloy

The entire experimental program was designed to obtain higher mechanical strength values of the studied alloy, combining hot and cold plastic deformations with solution treatments. For this, in the final experimental program stage, several parameter variants (two different heating temperatures and two holding times) were tested in two main directions—V-1 and V-2—as a possible way to increase the biomaterial’s strength, coupled with low Young’s modulus, while maintaining the bcc structure’s good mechanical processability.

**Figure 2:**
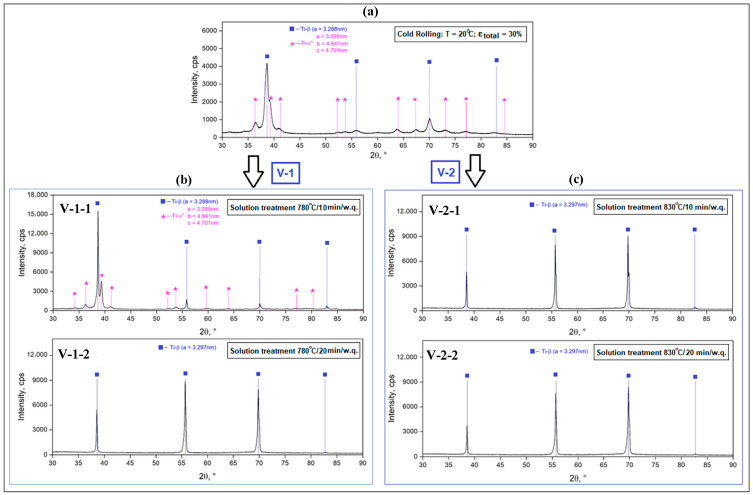
XRD spectra corresponding to: A-3, the cold rolled sample at 20 °C with ε_total_ = 30% (**a**); V-1, solution treatment at 780 °C/10 and 20 min/w.q. (**b**); V-2, solution treatment at 830 °C/10 and 20 min/w.q. (**c**).

Considering the XRD spectra for the A stages, samples A-1 and A-2 attest only to the presence of the β-metastable phase, which is understandable considering the high processing temperatures (950 °C and 820 °C, respectively), which are above the β-transus temperature. The four intense β-phase diffraction peaks are (110)_β_, (200)_β_, (211)_β_ and (220)_β_, and correspond to the bcc crystallographic structure and space group Im3m, labeled according to ICDD no. 04-002-8708 [46]. The β-phase lattice parameter is a_β_ = 3.297 Å (0.3297 nm). For sample A-3 (Figure 2a) corresponding to the cold rolling stage, apart from the high peaks of the β-metastable phase, the XRD spectra show several small peaks corresponding to the α″-martensitic phase, which appear as a result of the stress-induced martensitic transformation β⟶α″ in a proportion of approximately 8.9%. The resulting lattice parameter for the β phase is a_β_ = 3.288 Å (0.3288 nm). For the α″-martensitic phase (orthorhombic system—space group Cmcm), the determined lattice parameters are as follows: a_α_ = 3.095 Å; b_α_ = 4.941 Å; c_α_ = 4.701 Å.

Regarding the samples corresponding to stages V-1 (Figure 2b) and V-2 (Figure 2c), all peaks appearing in the XRD spectra only correspond to the β-metastable phase, which attests to the fact that the α″-martensitic phase, formed in the previous cold rolling stage, disappeared from the microstructure by dissolving in the β solid solution. For the 780 °C/10 min variant, however, α″ martensitic phase traces appear in the acquisition data, at an insignificant proportion of 0.1%, with the holding time being too short for a total dissolution. For the remaining variants, 780 °C/20 min and 830 °C/10 and 20 min, the α” phase disappears completely from the XRD spectrum, a sign that it has completely dissolved in the β-solid solution.

Due to the structural evolution analysis of the studied alloy, it is necessary to consider the ability to form a stable solid solution in the alloy, in this case the bcc metastable beta solution. There are two complementary ways of evaluating such an ability: one refers to the Mo_ech_ parameter and the second refers to the enthalpy of mixing (ΔHmix).

The first parameter—Mo_ech_—evaluates structure’s β-stabilization level by the titanium alloy chemical components. For the studied alloy, using the calculation formula from [13,47] results in a value of 10.88% for Mo_ech_, which is at the lower limit of the 10–30% range necessary to obtain a metastable, heat-treatable and deeply hard titanium alloy structure [13].

Regarding the second parameter, the enthalpy of mixing (ΔHmix), it is considered crucial for defining the specific solid solution formation properties [48]. Based on [49,50,51,52], if the enthalpy of mixing has negative values, especially for the bcc phase, an increase in mechanical properties, such as strength, can be expected. This negative enthalpy of mixing means that the interactions between titanium atoms and specific alloying element atoms are much stronger than those between the pure elements present.

Specifically, for the studied alloy, the ability to form a stable β solid solution can be expressed by the enthalpy of mixing, ΔHmix, which can be calculated with Miedema’s model [53] using the following formula:

(1) $$\Delta H\;mix=4\sum_{i=1;j=1}^{n}x_{i}x_{j}{\Delta H}_{i-j}^{mix}\cdot \;(i\neq j)$$

In this formula, $x_{i}$ and $x_{j}$ represent mole fractions of elements i and j, considered one-by-one from the combination of total elements in the alloy, while ${\Delta H}_{i-j}^{mix}$ represents the enthalpy of mixed i and j elements in a binary pair. The molar fractions of elements i and j, considered in binary pairs, are calculated based on the corresponding molar masses [g/mol], relative to a quantity of 1000 g [53], while for the related ${\Delta H}_{i-j}^{mix}$ values the data from [54] are used. Next, using references [54,55,56,57,58,59], ΔHmix = −8.7 kj/mol for the studied alloy. Zhang et al. [60] found that in order to form a complete solid solution, ΔHmix should generally be between −10 and 5 kJ/mol. For the present case, the resulting negative mixing enthalpy is a sign of stable β-solid solution formation.

Considering that all used alloying elements (niobium, tantalum, zirconium), except oxygen, have an atomic radius close to or larger than that of titanium, a substitutional type of the solid solution results, whose formation is supported by the empirical Begard law [61,62]. The ordinary lattice parameter for an unalloyed Ti-β phase is around a_β_ = 3.282 Å (COD 9,012,924 file). From the XRD analysis, the obtained solid solution’s lattice parameter increases to a_β_ = 3.297 Å due to the dissolved alloying elements. In addition, the atom size difference parameter—δ—of the alloy can be determined, as proposed by [60,63] to have the following formula:

(2) $$\delta =\sqrt{\sum_{i=1}^{n}c_{i}{\left(1-\;\frac{r_{i}}{\sum_{j=1}^{n}c_{j}r_{j}}\right)}^{2}}$$

where *c_i_*—atomic fraction of element *i*, *r_i_*—atomic radius of element *i* and *n*—total number of constituent elements. For the present case, considering Ti-24Nb-3Zr-1Ta-0.61O (at %), and chemical elements’ atomic radii $r_{Ti}\;$ = 1.462 Å, $r_{Nb}\;$ = 1.46 Å, $r_{Zr}\;$ = 1.603 Å, $r_{Ta}\;$ = 2.09 Å and $r_{O}\;$ = 0.73 Å, δ = 0.06026 results from calculus, which represents an atom size difference parameter that is not too large, a sign that the crystal lattice is not very strained by alloying elements. These results can also be considered a good sign of β-solid solution stability.

**Figure 3:**
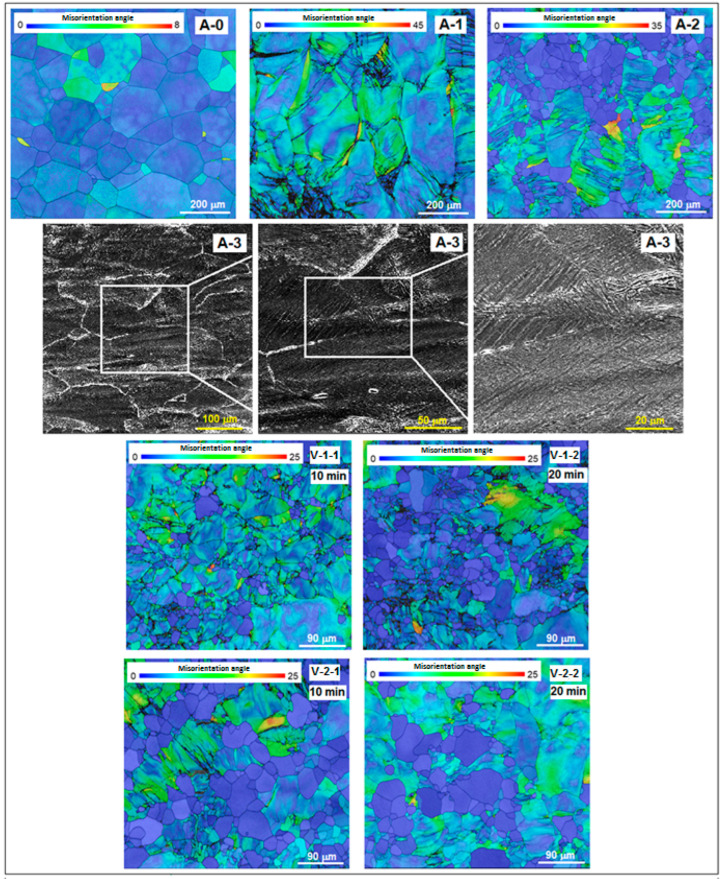
SEM maps of misorientation angle distribution corresponding to: A-0, as-cast initial state; A-1, Hot Rolling (950 °C/ε = 40%/a.c.); A-2, Solution Treatment (820 °C/30 min/w.q.); A-3, CR (20 °C/ε = 30%); V-1-1, ST (780 °C/10 min/w.q.); V-1-2, ST (780 °C/20 min/w.q.); V-2-1, ST (830 °C/10 min/w.q.); V-2-2, ST (830 °C/20 min/w.q.).

Figure 3 presents SEM-EBSD images combined with misorientation angle distribution maps of the studied alloy corresponding to stages A and V.

For the as-cast initial state A-0, a microstructure with equiaxed polygonal homogeneous grains of β-phase can be observed. Due to repeated re-melting procedures during the synthesis process, the strained grain areas are reduced.

For hot rolling sample A-1, the microscopic image shows a large number of heterogeneous β-phase grains which are visibly strained due to the hatched areas. Dimensionally, they are coarser than those from the as-cast state (A-0) due to the high rolling temperature.

For sample A-2, processed by solution treatment (820 °C/30 min/w.q.), the representative image from Figure 3 shows some persisting hatched areas with large β-phase grains, which are deformed and have a strained character, combined with new areas of much smaller and homogeneous new recrystallized grains. At this stage, only some of the previous strained grains transform into recrystallized ones.

For the cold rolled sample A-3, there is a series of three images with increasing magnification showing a highly deformed microstructure. Around the β-deformed grains, the white network indicates the presence of α″ martensite, which is formed due to the stress-induced martensitic transformation, a fact also attested by the XRD spectra discussed previously (Figure 2a).

For the two samples of the V-1 solution treatment variant (780 °C/10 min and 20 min/w.q.), the microscopic images show a mixture of some undeformed equiaxial grains with a slight dimensional non-uniformity combined with some strained grains, persisting from the previous cold rolling process. The variant with a longer holding time (20 min) indicates the same microstructural character, but with coarser grains than that with a duration of 10 min; the number of newly recrystallized grains is also larger. Concerning the two samples of the V-2 solution treatment variant (830 °C/10 min and 20 min/w.q.), the microscopic images show a morphological character similar to the V-1 variant, but the recrystallized grains, dimensionally more homogeneous, are visible coarser than for V-1. However, the refinement of grains from the as-cast stage to the final solution treatments is evident. Table 1 indicates the β-phase grain average dimension evolution, for the entire experimental program using the ASTM grain size measurement standard. Compared to the initial stage A-0, the grain dimension diminution for V-1 is about 74% for the holding time of 10 min, and 69% for 20 min; as for the V-2 variant, the diminution is about 60% for 10 min and 58% for 20 min, respectively.

### 3.2. Analysis of the Alloy Mechanical Property Evolution

**Figure 4:**
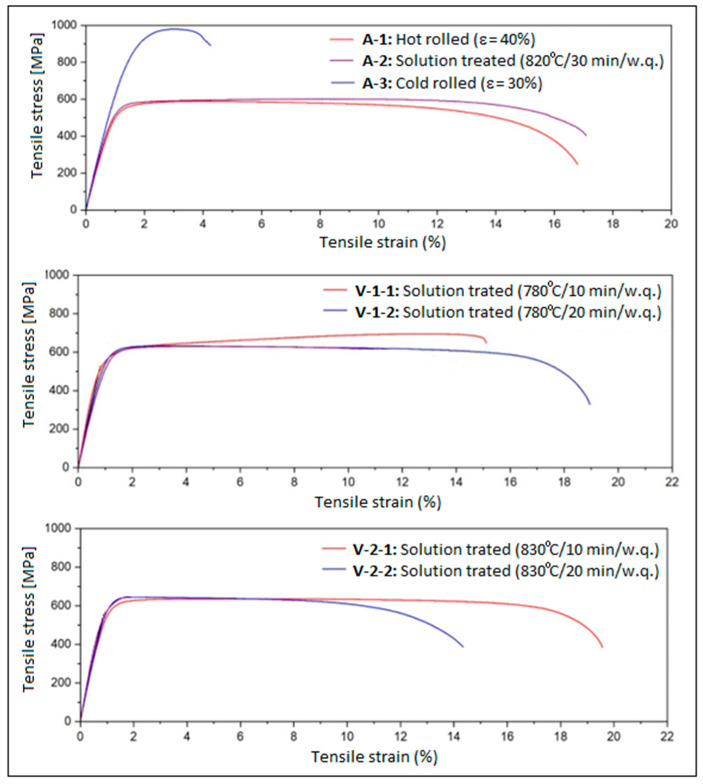
The strain–stress curves for: A-1, Hot Rolling (950 °C/ε = 40%/a.c.); A-2, Solution Treatment (820 °C/30 min/w.q.); A-3, CR (20 °C/ε = 30%); V-1-1, ST (780 °C/10 min/w.q.); V-1-2, ST (780 °C/20 min/w.q.); V-2-1, ST (830 °C/10 min/w.q.); V-2-2, ST (830 °C/20 min/w.q.).

**Figure 5:**
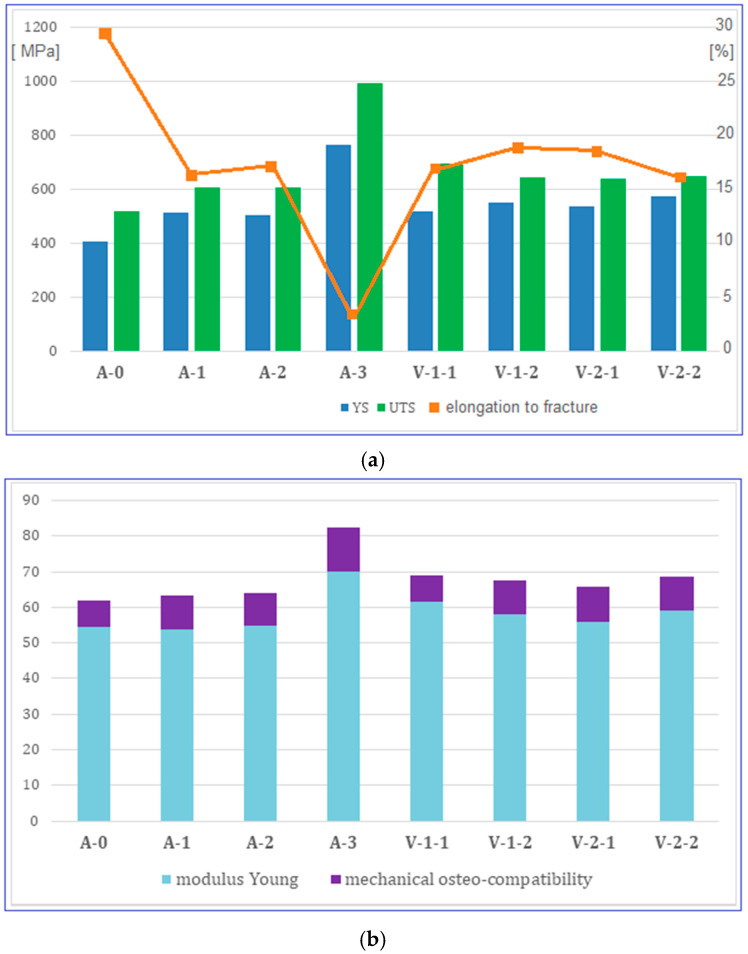
Evolution of the studied alloy mechanical properties: (**a**) value pairs for ultimate tensile strength (MPa)—UTS and yield strength (MPa)—YS; (**b**) value pairs for Young’s modulus (GPa)—E and YS/E—mechanical osteo-compatibility parameter. A-0, as-cast initial state; A-1, Hot Rolling (950 °C/ε = 40%/a.c.); A-2, Solution Treatment (820 °C/30 min/w.q.); A-3, CR (20 °C/ε = 30%); V-1-1, ST (780 °C/10 min/w.q.); V-1-2, ST (780 °C/20 min/w.q.); V-2-1, ST (830 °C/10 min/w.q.); V-2-2, ST (830 °C/20 min/w.q.).

The evolution of the mechanical properties of the alloy was highlighted by performing an analysis based on tensile tests for all processed alloy stages. Figure 4 shows the strain–stress curves obtained after the applied tensile tests, and Table 2 indicates the resulting values of the analyzed mechanical properties: ultimate tensile strength UTS (MPa), yield strength YS (MPa), Young’s modulus E (GPa), elongation at fracture ε (%), and mechanical osteo-compatibility parameter YS/E. Therefore, using the data in Table 2, the diagrams in Figure 5 were determined, which show the evolution of the resulting mechanical characteristics for all tested stages.

A gradual increase in YS and UTS can be observed from stage A-0 (casting) to A-3 (CR), after which the values decrease to a ceiling, at approximately similar values, for stages V-1 and V-2. The elongation to fracture values evolves inversely to those corresponding to the strength, with a value ceiling of approximately 17% also recorded for stages V-1 and V-2.

What is encouraging is that relatively low values were obtained for Young’s modulus, for variants V-1 (20 min) and V-2 (10 and 20 min), of approximately 56–59 GPa, with the exception of variant V-1 (10 min), which, due to the presence of α” phase martensitic traces, presents a slightly higher Young’s modulus value.

These results, namely suitable strength, low Young’s modulus and sufficiently high elongation at fracture, could confirm the success of the proposed experimental program action, improving the mechanical characteristics by achieving the following: (1) establishing a particular chemical composition that induces solid solution hardening through β-stabilizing elements, which ensure stability of the beta solid solution, and through the interstitial element, oxygen, which contributes to solid solution hardening; (2) developing an appropriate sequence of thermo-mechanical processing stages, interspersing plastic deformations with solution treatments and obtaining final mechanical properties that are optimal for the bone implantology field.

Comparing the V-1 and V-2 results with those reported in other related works (detailed below), the following can be found: compared with well-known Ti-6Al-4V (a), an impressive decrease in Young’s modulus by almost 50 GPa is obtained, and compared with similar β-phase titanium alloys (b–f), the decrease is almost 10 GPa. The UTS values fall within a median value compared to those selected for comparison, which can be considered to cover the result related to hard bone tissue values.

(a) Ti-6Al-4V (wt.%): UTS = 1100 MPa; E = 120 GPa [48];
(b) Ti-35.3Nb-7.1Zr-5.1Ta (wt.%): UTS = 500 MPa; E = 63 GPa [10];
(c) Ti-41.1Nb-7.1Zr (wt.%): UTS = 500 MPa; E = 65 GPa [18];
(d) Ti-30Nb-12Zr-5Ta-2Sn-1.25Fe (wt.%): UTS = 705 MPa; E = 55 GPa; εf = 11% [64];
(e) Ti-15Mo-5Zr-3Al (wt.%): UTS = 820 MPa; E = 78 GPa; εf = 11% [48];
(f) Ti-25Nb-17Ta-1Fe-0.25O (wt.%): UTS = 851 MPa; E = 60 GPa [19];
(g) Ti-36.5Nb-4.5Zr-3Ta-0.16O (wt.%): UTS = 640 MPa; E = 56 GPa; present work.

Special emphasis is placed on mechanical osteo-compatibility parameter, also determined and indicated in Table 2. Generally, the mechanical osteo-compatibility parameter of a metallic biomaterial is defined as the ratio of yield stress to Young’s modulus of elasticity (YS/E) [48]. The higher the YS values and the lower Young’s modulus, the better the mechanical osteo-compatibility will be. For the present case, the value of around 9.6 obtained for the final stages V-1 and V-2, balanced with the low Young’s modulus values (Figure 5b), is not a defining value in and of itself, but could highlight a direction to follow.

## 4. Conclusions

A previously untested TNTZ-type alloy with oxygen (Ti-36.5Nb-4.5Zr-3Ta-0.16O wt.%) was subjected to a thermo-mechanical processing program.

Through X-ray diffraction and SEM microscopy, it was demonstrated that a structure only consisting of stable β-solid solution was obtained, compared to the initial cast sample, with a grain size significantly reduced by approximately 60%.

The stability of the β-solid solution was analyzed through the enthalpy of mixing of the alloy—ΔHmix—and through the alloy’s atom size difference parameter—δ.

After performing tensile tests, the determined mechanical characteristics indicated suitable results: around 550 MPa for YS, 650 MPa for UTS and 17–18% for elongation to fracture, associated with a low Young’s modulus of about 56–58 GPa.

## Figures and Tables

**Table 1:** Grain size average evolution (µm) of the β-phase corresponding to the experimental samples; the reference value corresponds to the initial cast sample A-0 (a.c.—air cooling; w.q.—water quenching).

Sample	Processing Details	Average Grain Dimension of β Phase (µm)	Reduction of Granulation (%)
A-0	As-cast	204.3 ± 2.4	Reference value
A-1	Hot Rolling: 950 °C/ε = 40%/a.c.	316.4 ± 3.2	-
A-2	Solution Treatment: 820 °C/30 min/w.q.	76.7 ± 1.2	62.45%
A-3	Cold Rolling: 20 °C/ε = 30%/a.c.	-	-
V-1-1	Solution Treatment: 780 °C/10 min/w.q.	52.9 ± 0.8	74.11%
V-1-2	Solution Treatment: 780 °C/20 min/w.q.	63.8 ± 0.9	68.77%
V-2-1	Solution Treatment: 830 °C/10 min/w.q.	82.7 ± 1.8	59.52%
V-2-2	Solution Treatment: 830 °C/20 min/w.q.	86.2 ± 1.6	57.80%

**Table 2:** Mechanical properties determined for the studied alloy: UTS (MPa)—ultimate tensile strength; YS (MPa)—yield strength; E (GPa)—Young’s modulus; ε (%)—elongation to fracture; YS/E—mechanical osteo-compatibility parameter.

Sample		UTS (MPa)	YS (MPa)	E (GPa)	ε (%)	YS/E
A-0	Test 1	537.16	412.54	55.17	35.48	
	Test 2	495.40	398.94	53.51	22.46	
	Average	516.28 ± 20.88	405.74 ± 6.80	54.34 ± 0.83	28.97 ± 6.51	7.46
A-1	Test 1	588.09	498.31	53.30	16.79	
	Test 2	622.26	524.50	54.23	14.61	
	Average	605.18 ± 17.09	511.41 ± 13.10	53.77 ± 0.47	15.70 ± 1.09	9.51
A-2	Test 1	600.51	499.86	56.39	17.08	
	Test 2	611.19	504.13	53.62	16.99	
	Average	605.85 ± 5.34	502.00 ± 2.13	55.01 ± 1.39	17.04 ± 0.04	9.13
A-3	Test 1	978.65	731.13	69.81	4.25	
	Test 2	1009.20	796.57	70.35	3.03	
	Average	993.93 ± 15.28	763.85 ± 32.72	70.08 ± 0.27	3.64 ± 0.61	12.41
V-1-1	Test 1	694.72	517.46	59.21	15.10	
	Test 2	697.27	521.56	63.84	18.32	
	Average	696.00 ± 1.27	519.51 ± 2.05	61.53 ± 2.32	16.71 ± 1.61	7.41
V-1-2	Test 1	632.02	541.97	57.41	18.94	
	Test 2	657.34	561.17	58.65	18.04	
	Average	644.68 ± 12.66	551.57 ± 9.60	58.03 ± 0.62	18.49 ± 0.45	9.51
V-2-1	Test 1	636.34	541.11	57.94	19.57	
	Test 2	644.16	533.48	54.21	16.62	
	Average	640.25 ± 3.91	537.30 ± 3.82	56.08 ± 1.87	18.10 ± 1.48	9.58
V-2-2	Test 1	645.34	577.52	61.52	14.35	
	Test 2	651.63	567.34	56.69	17.98	
	Average	648.49 ± 3.14	572.43 ± 5.09	59.11 ± 2.42	16.17 ± 1.82	9.68

## Data Availability

The original contributions presented in this study are included in the article. Further inquiries can be directed to the corresponding author.
